# Quality of life whilst ageing in opioid agonist treatment: a narrative analysis

**DOI:** 10.1186/s12889-025-22438-4

**Published:** 2025-03-26

**Authors:** John Todd-Kvam, Gustavo Sugahara, Ashley Elizabeth Muller, Thomas Clausen

**Affiliations:** 1 Hospital of Southern Norway, Sørlandet Sykehus HF, Postboks 416 Lundsiden, Kristiansand, 4604 Norway; 2 Norwegian Centre for Addiction Research, University of Oslo, Postboks 1039 Blindern, Oslo, 0315 Norway; 3 Trondheim Municipality, Trondheim, Norway

**Keywords:** Opioid agonist treatment, Quality of life, Ageing, Social relationships, Victimisation, Narrative analysis

## Abstract

**Background:**

Norway has an increasing number of ageing opioid agonist treatment (OAT) patients, with 44% of the 8200 Norwegian OAT patients over 50 in 2023.

**Methods:**

This study examines the narratives of ageing OAT patients through semi-structured interviews with twelve patients who had been in OAT for 10–20+ years. We used narrative analysis to understand what they experience as important in enhancing or diminishing their quality of life as they age.

**Results:**

Positive relationships, treatment, and stable housing were narrated as enhancing quality of life, while loneliness and isolation, memory problems, comorbidities, and victimization were narrated as diminishing it.

**Conclusion:**

Patients experience OAT as both lifesaving as well as potentially limiting their life-quality, illustrating the inbuilt dilemmas of OAT. The study suggests an age-informed treatment model and identifies three thematic implications for practice and further research (on memory issues, victimisation and network-building).

## Background

Opioid Agonist Treatment (OAT) has prolonged lives of patients compared to those not in treatment, to the extent that 44% of Norwegian OAT patients are now over 50 [1]. This is both a success and a challenge, in that an ageing cohort of patients creates new needs in terms of research knowledge and its clinical application. We need to know more about these ageing patients’ health needs, quality of life, and the health care system’s capacity to assess and meet these needs. Two recent reviews have identified a dearth of studies examining tailored treatment services for older opioid users and older OAT patients [2, 3].

Norwegian OAT can be seen as a ‘critical case’ [4], with strategic importance for ageing and OAT, both because of its ageing cohort of OAT patients and because of the state’s capacity and will to help (and intervene with) its polity. In terms of *capacity*, Norway is topped only by Switzerland and Liechtenstein in healthcare expenditure per capita in Europe and is amongst the biggest spenders on social protection as a percentage of GDP [5, 6]. Regarding *will*, the Norwegian state has been assessed as willing to intervene significantly in the lives of its polity to effect change/rehabilitation, including for example the use of indeterminate prison sentences [7, 8]. Norway’s ambitious welfare system is often a reference worldwide as an example of in-depth service provision. In this sense, efforts to continue promoting OAT patients’ quality of life as they age, and the knowledge acquired during this process, should also serve a broader array of patients globally.

Within a European context, Norway adopted OAT relatively late. Upon its establishment as a national program in 1998, OAT was intended as the last treatment option for those who had exhausted other treatment options, all abstinence-based [9]. Initially restrictive inclusion criteria meant patients had to be at least 25 years old, dependent on heroin for “several” years and agree to aim for full abstinence of all illegal substances. OAT has been steadily becoming less restrictive since 2007 [10], and is now often the first, rather than the last, treatment option for opioid dependence [11], and the initial model has been replaced by a more harm reduction/low threshold approach. It is intended as a long-term treatment, including up to lifetime, and while safety/control mechanisms remain, treatment discharge shall not be used as a response to breaking treatment rules. The first national treatment guidelines were issued in 2010, and the new guidelines introduced in May 2022 focus on more individually tailored treatment, more user participation and less formal control. Currently treatment coverage is estimated at over 70%, which is among the highest in Europe [12] and retention is high [13]. Overall, Norwegian OAT aims for extensive treatment coverage, with a comprehensive model within one system that relies on collaboration between services to enable differentialized treatment.

### OAT, quality of life and ageing

As OAT successfully prolongs life, and as opioid used disorder itself is increasingly recognized and treated within a chronic care model, patient-centred and patient-reported outcomes such as preservation of daily functioning and improved quality of life are important indicators of success in addition to reductions in opioid use and treatment retention [14].

Quality of life is a thoroughly operationalized term when measured quantitatively. A widely-used definition is “individuals’ perceptions of their position in life in the context of the culture and value systems in which they live and in relation to their goals, expectations, standards and concerns” [15]. Quality of life should, it is argued [16], be included as a standard outcome in studies of treatments for substance use disorders, given that subjective experiences of addiction and experiences of treatment (including potential iatrogenic effects) impact quality of life and that quality-of-life appraisals extend across multiple domains; this argument has also been made specifically for OAT [17].

Despite extensive psychometric development across multiple instruments, there are few consistent predictors, or even associations, of high quality of life among the general population or OAT patients beyond psychiatric comorbidities and overall mental health status [18–20]. As a subjective measure, quality of life is impacted by factors individuals deem important, and importance-assigning is crafted in cultural contexts (including overarching expectations about ageing along with specific cultural contexts of being in OAT).

Among Norwegian OAT patients, developing abstinent social networks [21], participating in group exercise [22] and improvements in housing, leisure and finances [23] have all been associated with enhanced quality of life in relatively small samples when measured quantitatively. Reduced social contact appeared to reduce quality of life in one of these samples [24] – an important issue since patients are often encouraged to withdraw from substance-using networks. Some OAT patients (from a younger sample) also try to combine treatment with illicit drug use and might end up in a liminal position, both feeling controlled by OAT whilst “simultaneously trying to trick them” [25]. One of the few studies on the life-situation of older OAT patients in Norway [26]found patients experienced stigmatization, isolation and financial difficulties. Understanding more about how these issues are experienced as OAT patients age is important.

### Knowledge gaps

Qualitative research has allowed OAT patients themselves to emphasize salient domains for their own quality of life [e.g. 19]. We are, though, aware of little research on the distinct experiences of older OAT patients´ quality of life. Selseng [27] describes how Norwegian welfare employee categorizations of clients needing substance use disorder treatment as young or old influenced recommendations and referrals to treatment, expectations of behaviour and success, and even assumptions of agency and responsibility (Meyers [28] notes similar thinking about adolescence as a ‘critical window’ for treatment). Existing research also highlights that older OAT patients in Norway are a population with psychiatric and somatic comorbidities and diverse needs, whilst also highlighting that “knowledge in this area is poorly developed and that the pilot study points therefore to a strong need for further research” [29]. Bartoszko [30, 31] notes that living with addiction and in OAT affects social and psychological ageing too. Given we have some research evidence for negative attitudes towards ageing and older persons in need of treatment, we need then to explore further OAT patients’ experiences with ageing, including the specific intersections of ageing with domains relevant to quality of life. Intersectionality [e.g. 32] and life-course perspectives [33] are as such important theoretical framing for our analysis. This is because intersectionality helps bring to light how membership of different categories (e.g. gender, age, income/class, race, health status) will not act separately and in isolation from one another but rather build upon and potentially reinforce one another [34]. For the purposes of our analysis, many of our informants experience an intersection of being female AND of being an OAT patient AND of being older. Regarding the life-course perspective, this helps us understand and contextualise how people experience different stages and phases of life (for example experiencing bereavement, becoming a grandparent). It is also useful when considering recommendations for practice, in that treatment needs may evolve over the life-course, including regarding OAT.

## Methods

### Aim

Our aim is to explore how older Norwegian OAT patients narrate their quality of life as they age *as OAT patients* to improve knowledge for this specific group and to draw out implications for treatment services. We analyse the narratives of a small sample of patients to better understand what they experience as important in enhancing or diminishing their quality of life. This article flows from a larger study aiming to improve understanding of ageing opioid and substance users to improve treatment and quality of life.

### Data collection

To meet inclusion criteria, patients had to be more than 50 years old, and with at least 4 years of experience in OAT. The informants were recruited purposively in cooperation with the user organisation proLAR Nett, who disseminated information on the project to their network of OAT patients, answered questions on the study and then put prospective informants in touch with author one. proLAR Nett also contributed to the development of the interview guide. The interviews were conducted and coded in Norwegian before being translated for inclusion in the article. Both native English and Norwegian speakers were involved in the analysis and writing up of the findings.

Author one interviewed 12 patients; 10 women and 2 men. Despite ages ranging from 50 to 69, informants tended to have entered OAT at around 40 years old. Nobody withdrew or was excluded from the study. With all but one informant having over 15 years in OAT, there is also a cohort effect in that these patients carry with them experience of the earlier iteration of OAT which was more focused on safety measures. Table 1 displays brief aggregate characteristics:

**Table 1:** Descriptive characteristics of informants

	*N*
**Age**	
50–54	5
55–59	3
60–64	1
65–69	3
**Time in OAT (years)**	
10–14	1
15–19	4
20+	7

Interviews lasted between 30 min and 1 h 45 min and were conducted over the telephone/Zoom. Informants were located in northern, mid and southern Norway and from urban and rural locations – important given geographic variation in OAT [35, 36]. The informed consent document was tested for accessibility and provided to prospective informants. It was submitted via an online form direct to an encrypted university server. The interviews were conducted with a ‘confluent’ interaction style [37], prioritizing rapport and positive atmosphere, but potentially avoiding confrontational or difficult questions. This approach was important for ethical reasons (patients may have experienced violence and trauma), and for methods reasons (avoiding fact-checking and allowing people to tell their stories).

The interviews started with an explanation of the project’s goals of understanding more about ageing and OAT, before asking an open question that gave informants the opportunity to share as much/little about their background and stories as they wanted. Further topics included:

- experiences with OAT (overall view of OAT, practicalities around treatment like collection and current dosage, positive/negative experiences, relationship with treatment personnel, unmet needs, thoughts about being older in OAT).
- physical health (changes over time, untreated problems, worries about pain management).
- mental health (changes over time, untreated problems, issues with cognition or memory).
- what is important in life (social relations, position in today’s society, feeling of belonging).
- finances and housing.

### Using narrative analysis

Our overarching aim is to bring out stories and experiences that seemed to matter to our informants and their perceived quality of life. We see these stories as being constructed in the interaction between interviewer and interviewee, in the context of an interview about OAT, health and quality of life. As Frank [38] argues: “people’s stories report their reality as they need to hear it, as well as reporting what they believe their listeners are prepared to hear”. Or, put another way, what we analyse are selected stories the informants chose to share in this particular context [39].

In terms of using narrative analysis, Riessmann [40] states it “allows for systematic study of personal experience and meaning: how events have been constructed by active subjects.” Adding an extra dimension to this, De Fina [41] argues “when storytellers tell a story, particularly one in which they were participants, they are simultaneously building their own and others’ identities in the story world and their identity in the storytelling world.” That is, there can be two levels of identity-work being accomplished – who the informant is/was at the time of the story and who the informant wishes to be in relation to the interviewer. De Fina further argues informants may also reproduce and recirculate shared representations (in this case potentially about OAT patients, particular medicines, OAT itself, and about ageing and old age). Frank [38] argues similarly that our sense of self is constrained by the resources available to us when we tell our stories, as well as the stories other people tell about people like us. In terms of our overarching analytical approach, we employed thematic narrative analysis [42]. In essence, this means that the first ordering principle of our analysis is *what* is being said, as opposed to structural narrative analysis, which places primary emphasis on *how* stories are told, and to the broader dialogic narrative analysis, which focuses on stories as interactionally produced and performative [38, 42]. The approaches are not mutually exclusive [43], so we use insights from structural and dialogic approaches, albeit under an overarching thematic framework.

### Coding

An initial set of codes was created by analysing relevant literature on OAT and ageing in NVivo, before being applied to patient interview transcripts. The codes were then further developed and refined (e.g., the addition of victimisation). The issues we interpreted as being of particular salience were then grouped into four overarching domains commonly explored when operationalizing quality of life: social relationships, physical and mental health, treatment experience and environmental/contextual factors[e.g. 44, 45]. Under each of these domains, key themes are then explored.

### Ethics

The project received ethical approval from the Regional Committee for Medical and Health Research Ethics South East C (ref. 28848). Given informants access a service that can be experienced as stigmatising and as having a control aspect, ensuring voluntary participation is both important and potentially challenging. Recruitment via user organisation, being clear participation/refusal would in no way be linked to treatment and the positionality of author one (who conducted the interviews) as working outside OAT and non-Norwegian may have been helpful in alleviating potential concerns, including of power asymmetry [46].

Research with vulnerable groups often involves difficult topics, including trauma, health problems and lack of hope. Consideration of participant distress is therefore important. We ensured each interview concluded with a ‘de-brief’, allowing the participant to air their reactions, listen to and empathise with any feelings of distress [47].

The sound files and subsequent transcripts are stored in encrypted form on the University of Oslo’s platform for sensitive data, and data protection approval was granted by SIKT, the Norwegian Agency for Shared Services in Education and Research (ref. 420282).

Regarding the ethics of analysis/interpretation, Squire [48] highlights ethical problems regarding presenting large amounts of data, for example by presenting whole life-stories, because anonymity becomes difficult to guarantee. We therefore present only small excerpts from our interview data, editing identifiable details (e.g. using pseudonyms, removing highly specific health problems and locations).

## Results and analysis

We structure this section across four domains (social relationships, physical and mental health, treatment experience, and environmental/contextual factors), analysing the links between those domains and ageing in OAT. The discourse on ageing among patients largely adheres to a biomedical viewpoint. This framework typically equates ageing with senescence, framing it as a series of issues and obstacles for individuals and the wider community. Patients contrasted their experiences with both ‘normal people’ and a perceived ‘normal ageing process,’ whilst also speaking of friends and acquaintances who had died young. At the same time, they engaged in conjecture about their own future as older adults, arguably indicating a hesitance to self-identify with this stage of life.

### Social relationships

#### The positive influence of friends and family

Social relationships are key in understanding the individual experience of ageing, marking new ‘life stages’ [49] whether it be through new relationships (e.g. becoming a grandparent) or losing old ones (e.g. becoming a widow, being advised to cut off drug-using friends). Our informants narrated positive experiences with friends and family, whilst others told stories of loss and loneliness. In terms of the positive:

Alice: *I have children who are really good at getting me out for a walk and also my daughter got me a little dog about a month ago. So*,* then I have some company and I get myself out for walks*,* I have some… some other things to do.* (Woman, late 50s)

This example is interesting in terms of its multidimensionality – Alice frames relationships with family as positive in and of themselves, and as a source of other positive influences – in this case physical activity through walking together and being given a pet dog, that provides both company and a reason to get out of the house[see 50 for more on this]. This small story is also a narrative about *how* social connection supports agency and autonomy[see 51], in that the presence of a positive family relationship helps Alice act agentically to take care of their new pet and to improve their own physical and mental health by ‘get[ting] myself out for walks’. Whilst this story might seem like a ‘normal’ narrative from someone who is getting older and have little to do with OAT, it is precisely this normalcy that is important to capture. We see both an ethical and practice-directed import in maintaining awareness that whilst OAT may remain an important aspect of older patients’ lives, their experiences are not necessarily defined by it, and indeed supporting positive social relationships will enhance their quality of life and potentially health-enhancing behaviours. This links to the treatment guidelines in Norway, which state that rehabilitation-related activity should start from the patient’s life situation and personal goals and how treatment services can support these [11].

Informants also told stories which included both elements linked to a history of addiction and universal child-rearing experiences:

Bex: *She is mamma’s darling. She is doing really well*,* but she was also on her way into it and started to use drugs. So then I said to Children’s Protective Services “now*,* now I have to get involved and I have to pick the place she’ll be sent*,* because you don’t have the competence*,* this is my area”. And CPS actually listened to me*,* which I thought was pretty decent*,* and she stopped using afterwards. […] I have tried to be there for her*,* the whole time*,* but it is like I say*,* they grow up and get busy with their own things. So now I am sitting alone again in a way. Eh*,* and that makes my anxiety eh*,* run away with itself sometimes.* (Woman, early 50s)

One aspect of Bex’s story is a familiar example of a parent of older children, feeling bittersweet as their child succeeds but becomes independent. Bex was proud to share her daughter’s success, whilst also expressing relief that whilst she too had started to use drugs, she was now drug-free and with a good, stable job. So this is both a narrative of a daughter’s success and a disnarrative [52] of what could have happened but didn’t – of a daughter following a parent into addiction. Such narratives of ‘making a break’ and avoiding an intergenerational cycle of addiction are likely to be important for all parents to whom such a narrative is available, but perhaps particularly for parents who are ageing and for whom generativity is of increasing importance [53]. Indeed, several informants told stories of children having done well and of having good contact with grandchildren; such narratives are, we contend, a positive opportunity for storytellers to exercise agency and align with existing cultural norms about parenthood, helping them construct a new part of their identity less defined by their status as an OAT patient. Concurrently, such stories highlight the relational aspect of ageing: when children grow up and become more independent, it has an impact on the parent’s life, self-perceived role and expectations with regards to their relationship, ushering in a different ‘life-stage’: awareness of this amongst OAT practitioners is important in maintaining constructive relationships with patients.

#### Loneliness, isolation and bereavement

Lack of social relationships was narrated as impacting negatively on quality of life. Loss and loneliness were highlighted as big challenges:

Bex: *Why are you so unstable then? Well*,* because you may not have anyone to trust*,* and after my husband died*,* I have become so alone. And I don’t have anyone - it’s not easy to build a network when you’re over 50.* (Woman, early 50s)

Again, the end of important family links is a milestone in the ageing process [54], implying a different life-stage. Given previous findings that developing abstinent social networks may have improved quality of life for OAT patients [21], the narration of network-building being difficult over the age of 50 is concerning and of relevance for treatment services. Bex here seeks to account for her instability through lacking anyone to trust and being ‘so alone’ following the death of her husband. This makes some intuitive sense, in that lacking a ‘backstage’ in which to vent frustrations or sense-check one’s experiences may lead to more volatile interactions when one finally does get to talk to someone (which may in turn increase isolation as OAT patients age). The use of the phrase ‘build a network’ is also interesting (as opposed to ‘make friends’ or ‘meet new people’) and perhaps speaks to a long period in OAT and with that exposure to social work talk about topics like network-building.

Bereavement was a recurring theme, with more informants talking about losing friends, partners and even children:

Christina: *To become so lonely - it’s the worst loneliness I’ve ever had - I have in fact many acquaintances*,* and friends*,* but I have pulled myself away completely. [later in the interview] There are so many who have died*,* right*,* I have lost all*,* my whole gang from childhood and my youth*,* all my close contacts. Those closest of the close.* (Woman, 60+)

Bex: *I have buried two children [I: that must have been hard] Yes*,* it was tough*,* much tougher than I thought. So*,* I have like lost both husband and children*,* I have experienced that. Two different griefs*,* you know.* (Woman, early 50s)

These events have a double impact, in that they create significant pain and grief whilst also increasing involuntary social isolation. Given connection with others may help cope with grief, the intersectional connection between ageing, loss and isolation for this group is of particular significance [55]. The notion of ‘pulling myself away completely’ chimes with Mayock and Butler’s [56] work with Irish OAT patients, who experience isolation and loneliness: “Among women in particular, negative self-regarding sentiments were frequently articulated and, while expressing a desire for friendship, companionship and intimacy, many appeared to have abandoned these aspirations.” (p. 146). In both the excerpts above, we see accumulation of loss over time, in that both informants talk about repeated experiences of bereavement. We know that living alone, poor health (including psychological distress) and bereavement are associated with loneliness in older people [57–60]. The intersectional connection between ageing, poor health, loss and isolation for this group is of particular significance, in that these factors are extra acute with OAT patients and may negatively reinforce one another. The problem of complicated grief (i.e. intense grief that impairs functioning and lasts for prolonged periods) may be an overlooked issue with older OAT patients, not least because risk factors include a history of mood or anxiety disorders, alcohol or drug abuse, and multiple losses [61, 62].

### Physical and mental health

#### Physical health comorbidities

This subsection focuses on descriptions of living with multiple physical health problems, with the biomedical perspective evident in this cultural context (i.e. a focus on somatic problems rather than, for example, having more time for hobbies/being a grandparent). Physical and mental health problems were a common topic when patients were asked about ageing in OAT both in terms of current problems and reflections about the future:

Dina: *I have a lot of pains in my body*,* a lot of somatic issues […] Because I had a malignant*,* aggressive tumour removed and received very strong chemotherapy*,* and had over 20 radiation treatments after that. That did something to me afterwards. When the treatment ceased completely*,* my body started to go weird. And there’s remembering things*,* but I have had a lot of UTIs*,* that’s of course something women of my age can get*,* a bit confused and forgetful and such*,* and that can be wrongly interpreted by OAT*,* because it is seen as using drugs instead of people thinking “you know*,* it could be somatic.” Because when I had lung problems*,* everyone accused me of being high. I fell asleep while I sat and talked to people*,* like now when I am sitting talking to you in the middle of a conversation*,* then I could fall asleep*,* and I think it was because my brain wasn’t getting enough oxygen at time.* (Woman, early 50s)

This excerpt highlights the overlapping range of issues some older OAT patients might deal with, and how Dina attributed a knock-on impact on how they were met by treatment personnel. Dina narrates an experience of being suspected of drug use because of somatic problems (lung issues) and problems which she attributes to ageing (memory problems, UTIs). The intersection of gender, age, and health status influences both provision of services and individual expectations and motivations. So, in addition to pain and uncertainty during and after cancer treatment, Dina narrates an additional problematic experience of assumptions of drug use from treatment personnel[chiming with 26]. The framing of OAT personnel as lacking trust is also explored in more detail in the specific section on treatment experience below.

Christina talked early in the interview about her childhood, noting sadly that:

*Yeah*,* so really – really I have tried to escape all of my past my whole life. But I’ve caught up with myself*,* caught myself as I’m becoming old and sick*,* when I started to get old.* (Woman, 60+)

This is a doubly ‘sad tale’ [63], in that not only does this person narrate a common story of fleeing from the past, but also reflects that this was ultimately unsuccessful. It is further noteworthy that this woman identifies herself as ‘old and sick’ in her late 50s. Later in the interview, when discussing reducing their OAT dosage as they get older, she noted:

*I don’t need so much because I’ve gotten this old - but also because I get so many other medicines: beta blockers*,* for high blood pressure*,* blood thinners*,* I take 12 different medicines every day. So they knock each other out*,* you know*,* […] lots of statins and stuff like that now.*

This is a further example of living with comorbidities, but also a small story where Christina emphasises that with increasing age comes an increasing scale of medication (‘12 different medicines a day’, ‘lots of statins and stuff’) – to the extent that they ‘knock each other on the head’. Separate interviews with treatment personnel also addressed this, with clinicians highlighting lack of knowledge about how medicines interact in older patients [X].

#### Physical mobility as agency

Accounts about activity and mobility varied, with some relating problems that limited mobility and quality of life:

Erica: *I am terrified of falling*,* because if I break my leg or ankle one more time*,* then there isn’t anything that can be done*,* I’ll lose the leg. So I have that hanging over me the whole time. So therefore last winter we got it sorted that they would come and deliver my methadone one day a week*,* but you can’t trust the home nurses either. Like*,* I wake up at 6am in a bad state and I have to wait until maybe 11*,* 11:30 before they come. I never know when they will come*,* right*,* […] and it’s the unpredictability I can’t manage. Then I can easily get aggressive when I am feeling bad*,* I get very*,* very nasty*,* well not nasty but I run my mouth off and am not easy to deal with*,* I get confrontational and behave inappropriately.* (Woman, 60+)

This account is, again, interlinked – a story about mobility problems because of an injured leg is linked to ongoing fear (of amputation), to being worried about leaving home when it is icy or snowy, and to becoming dependent on home-delivery of methadone. Erica here links these uncertainties to confrontational behaviour with the nurse who delivers her methadone. Another example of mobility problems was described as follows:

Alice: *What’s a real shame is that I have osteoarthritis in both hips. So I have problems with mobility and that’s very tiresome. And then the methadone*,* I gained nearly 50 kg. So… I am a bit overweight still.* (Woman, late 50s)

Here we can see how a combination of osteoarthritis and weight problems are attributed as impacting negatively on the Alice’s quality of life, albeit whilst asserting that she is taking steps to reduce her weight. There were also examples of comparison with other people of a similar age:

Bex: *I look at other 50-somethings and I think they look sporty and fit*,* right. And it*,* yeah*,* and I feel as if I am*,* yeah*,* feel like I’m an old 80-year-old.* (Woman, early 50s)

This is a narration of a sense of difference, of being less fit than others of a similar age (this may have extra resonance in the Norwegian cultural context where being physically fit and active carries significant symbolic capital [64]). However, other informants were more positive about their mobility, describing enjoying activities like climbing mountains:

Frankie: *For my 50th birthday*,* instead of a big party*,* because I don’t drink alcohol at all*,* I [completed an iconic mountain hike]. And now I have my 60th*,* what shall I come up with now? […] I think for my 60th I want to do something just for myself*,* just like I did for my 50th. Something I’ll remember for the rest of my life. Something as a reward for myself!* (Woman, late 50s)

Frankie’s story shows agency in both deciding *against* a large party and *to* engage in a strenuous but rewarding hike to celebrate a major milestone. This experience is related as having a positive impact, both through remembering it and through using it as inspiration to continue to take agentic decisions that are good for her and good to her (i.e., she sees herself as worthy of rewarding). So, whilst for some older OAT patients, their physical mobility is narrated as being associated with dependency and pain, for others it is framed as a source of positive life quality. In particular, we can see that lack of physical mobility is narrated as a source of difference from ‘other 50-somethings’ and narrated as a factor in negative interactions with treatment personnel.

#### Short-term memory problems

One of the key experiences from a health perspective was of losing short-term memory, with ten of twelve informants connecting memory problems in varying degrees to ageing, OAT, polypharmacy, and social situations:

Frankie: *I don’t know if it is to do with low metabolism or if it is time or if it is the OAT-medicine or what it is*,* but I struggle with my memory and so on… just the short-term thing*,* the long-term thing is totally fine.* (Woman, late 50s)

Erica: *I don’t know if it’s memory*,* at least I’m at a loss for words and fall out of things out a bit quickly. I don’t know if it has anything to do with the methadone - then maybe it’s for them to say “no*,* it’s your other medications as well.” Yeah*,* there are things like that I’m afraid to bring up*,* you know*,* because then it’s not just about*,* like*,* methadone anymore*,* then it’s the other medicines that do that. And I don’t want to be part of that. No*,* the medicines I have now*,* I will have them until I die*,* plain and simple. […] No*,* I just want it to be as good as possible until I am done with this life*,* I am tired.* (Woman, 60+)

Informants highlight the direct negative impact on quality of life, via narrations of not being able to remember what one has done that day (which could lead to harmful management of medicines) or falling out of conversations. Whilst we cannot make any firm conclusions regarding the veracity of our informants’ attributions here regarding long-term OAT and memory problems, that ageing OAT patients report short-term memory problems as impacting their quality of life is nonetheless important, even if iatrogenicity plays a limited role. There is also the potential that memory problems impinge upon agentic, quality of life-improving behaviours like socialising and being out in the world, as well as no longer being able to live independently:

Erica: *I’m terrified that one day*,* in not too long*,* I might have to move into a care home because I’m starting to get quite forgetful*,* I mess up a lot*,* I have to write down what I’m going to do and stuff like that […] So I write little notes then*,* just to try to remember. […] But I think it’s gotten better after I started training*,* now I’ve only been training for three months*,* but I notice a big difference.* (Woman, 60+)

This imagined future raises fears about independent living (commonly and sometimes exaggeratedly held beyond OAT patients), but Erica relates how starting to do more physical activity has helped with her memory problems. So, whilst there is the potential for short-term memory problems to isolate, there are alternative responses available, at least for some. Overall, though, this finding about short-term memory problems and the concerns they raise about loss of independence amongst older Norwegian OAT patients is important given the direct implications for quality of life (e.g. living with fear for the future, experiencing conversations as difficult to maintain) and for patients’ ability to manage increasingly complex prescription regimes.

### Treatment experience

There is a complex, two-way relationship between experience of OAT and quality of life (for example, dissatisfaction with medications or OAT in general has been reported as partly explaining reports of side effects [65]). Informants narrated a broad range of treatment experiences across often significant time in OAT:

#### When it works

OAT in Norway is based on cooperation between specialist health services, primary healthcare (mainly GPs) and municipal services (e.g., municipal substance use teams, home nursing care). For some, this cooperation has worked well:

Greta: *When my partner and I started in OAT we got a lot of good help and support*,* but I don’t know if it was the OAT system or if it was the [municipality’s] substance abuse team or if it was our GP*,* like it was the totality*,* but I have to say it saved us*,* it did.* (Woman, early 50s)

The experience of being ‘saved’ by OAT through an experience of seamless help and support is a textbook example of how such cooperation should work within OAT, and the narrator here making an evaluative affirmation that OAT added years to their lives (‘it saved us’). Others related experiences of having little contact with OAT, with treatment having been transferred to primary care services:

Harry: *I have very little to do with OAT (often referred to as the specialist health services part of the cooperation in OAT). You know*,* I was transferred over to my GP after only a few years. So the only thing I have with OAT is that once a year they ring me for one of those interviews – that is the only thing I have with OAT. All decisions are taken by my GP*,* he writes the prescriptions. So for me it works very well*. (Man, 60+)

This light-touch approach is narrated as working well for Harry, who has a good relationship with their GP and does not feel a need for regular contact with OAT (specialist health services). This story is as such an indication of the potential for a specific age-informed treatment model for older, more stable OAT patients that is lighter touch and helps support agency.

#### Too much turnover too little trust

Breaches in existing relationships with treatment personnel was narrated as a challenge by a number of informants. For example, Christina described her experience following two breakdowns in relationships with psychologists who changed jobs:

*Twice I have been left alone with my history*,* in a way*,* without getting any help to resolve it or to move on. To finish certain chapters. Do you understand what I mean? That - because they quit*,* I have never got anything - it’s just something that has been stirred up again and again*,* it’s like standing and stomping in molasses.* (Woman, 60+)

Similarly, Frankie discussed the difficulty of having to bring up painful, traumatic memories and experiences with new personnel:

*Like for me you have your story that you can’t bear to start explaining again and again. […] I can’t stand ripping all that up again*. (Woman, late 50s)

These experiences of retelling painful stories without being helped are particularly acute consequences of staff turnover, in that they risk re-traumatizing already vulnerable patients. There is also a temporal aspect to this, in that over half of our informants had been in OAT for over 20 years (and 4 of the remaining 5 over 15 years). Several informants narrate experiences of being let down as they age by staff that changed job. Frankie narrates such breaches as tiring as well as painful:

*I have had group meetings which gave me a sense of security. But then I had five different doctors in two years*,* and I lost my drive. It’s about trust again - build it again and again and again*,* and it is so… it’s so difficult. I feel I gave gone backwards a bit*,* so I have to roll up my sleeves and work. It’s tiring*,* I am starting to get exhausted by it.* (Woman, late 50s)

Informants here narrate a painful experience of re-telling stories and re-building relationships: treatment providers should carefully consider the therapeutic value of such conversations, particularly when long-term follow-up may be challenging. OAT treatment providers should manage change of staff better for this group, who may find it more difficult to rebuild trust and retell their stories, particularly if they have already felt required to do so multiple times already[see also 66]. This is important given OAT patients often have strongly negative relational experiences and because lack of trust is described as impacting how patients navigate their treatment (it also acts as a threshold to overcome in accessing services [67]).

The issue of trust was relatively varied. Whilst some informants explained they had solid relationships with primary treatment providers in OAT, and some emphasised their visiting nurse as a key trusted contact, others complained about a lack of trust:

I: Can you say a bit more about relationships with OAT staff? Are there people there you trust?

Alice: *No*,* nobody! [Okay*,* mmm] Not… none at all*,* because my experience is that every time I’ve asked them about something it has only made trouble.* (Woman, late 50s)

I: [After mentioning struggles with anxiety] Are you getting any follow-up, or someone you can talk to about the anxiety?

Bex: *No*,* there is really nobody in the system I trust enough to open my heart to. Because there have been too many disappointments*,* and a bit too many*,* how should I describe it*,* experiences of stigma.* (Woman, early 50s)

Informants here narrate experiences of mistrust that have built up over significant time in OAT and that create barriers to receiving the full potential of help from the treatment. Larsen and Sagvaag [68] have observed similar challenges regarding Norwegian OAT. Age was also used to make a point about lack of trust:

Frankie: *I seriously got more trust when I was 10 than now that I’m over 60. It’s a struggle. That there*,* that is really what tires me out most*,* that impacts my body*,* and it impacts eh. my psyche even more.* (Woman, late 50s)

Isla: *It is so tiresome to all the time be disbelieved*,* never be believed*,* everything is seen with negative eyes*,* so I have to say that I am terrified of the thought ‘what happens to me when I start to get old and start to need more help?’ I can barely do normal housework today.*

Once again, perceived lack of trust is narrated as tiring, both physically and mentally. Treatment providers should bear in mind older patients may have undergone what they experience as multiple breaches of trust both in other aspects of their lives and during earlier, more restrictive treatment approaches.

#### The potentially abnormalizing aspects of control

OAT in Norway may, for some, involve a significant element of control/safety measures, including supervised administration, urine testing, and limited ability to take medicine home. These can be demanding routines with an impact on patient´s lives. However, for others the system is more light-touch, without testing and with approval to take home up to two weeks’ worth of medicine. In terms of the lighter-touch approach:

Harry: *Yeah*,* I collect once a week and if I want to*,* I can get medicine for two or three weeks and it isn’t a problem. I take my medicine under supervision once a month. I take two urine tests a year*,* I decide myself when I take them. I’ve got it pretty good.* (Man, 60+)

For Harry, the ability to experience a significant amount of agency over how his treatment is managed is narrated as positive: both in terms of control over how often he collects his medicine but also when he delivers urine tests. Others narrate a more ambivalent relation to testing:

Frankie: *I have given morning-tests for 17 years and in these 17 years I have had zero problems. Sooo… then… I had decided that because of the mistrust from OAT that I would never ask to stop the urine tests myself. [*Note: requests to stop urine tests risk interpretation by some OAT personnel as implying interest in substance use*] […] Imagine if words were enough*,* but at the same time I’ve seen again and again that with a history of drug use you get mistrust. You can be accused of things you haven’t done; I have had kids through all this*,* and Child Protective Services have received reports of concern – I don’t know how many messages they’ve received – but every case has been dropped within a week. Because they know*,* they know. They have checked my urine tests*,* and they were ok. So*,* in a way the urine tests have been positive in that I can relax and not think about it. But then I can’t of course say that I wanted to continue the tests for over 17 years*. (Woman, late 50s)

This theme of urine tests providing a kind of security for those with children was reported by several informants and in separate interviews with treatment personnel. This is an example of how welfare help and control intersect, in that clean tests were narrated as crucial for managing the relationship with Child Protective Services and to enable continued care responsibility for children. Frankie went on to say:

*I said like to the OAT-woman (treatment provider) when she was collecting the urine test “Tell me something”*,* I said “are you planning to continue like this when I’m lying in a care home and… and use nappies and you squeeze out the pee from the nappy as well?”*,* I said to her. She got very strange after that. [laughs] Of course it was just a picture of us as elderly people. We will surely end up with nappies at some point as well. Are we really going to have the same mistrust our whole lives? Is it necessary? Is it necessary to put us through that?*

Frankie frames care homes and incontinence as negative and unavoidable features of old age (these are hardly OAT-specific fears, rather reproductions of broadly held ideas about ageing and old age). This story can be interpreted as a story about resistance against control and as an attempt by Frankie to normalise herself, putting herself in the same position as a stereotyped version of an older people. It is also a colourful way to make a point in the context of the interview situation about mistrust and whether urine testing is necessary for this older, generally more stable patient group. A similar point was made by Alice, who uses her age to reinforce a point about her experience of urine testing:

*I was awfully fed up with surveillance and arbitrary punishments*,* like restricted collection after a positive test for alcohol. Ehmm… monitored urine tests. Nearly 60 years old… why continue with it? I haven’t had a dirty test for anything other than occasionally for alcohol. Ehmm…. my husband was really sick*,* incredible amounts of pain*,* his [internal organ] burst*,* yeah*,* lots of pain. He was refused painkillers because he was in OAT. So*,* the final few weeks of his life he got pain management with 1000 mg dolcontin per day. Something he should have had many years before. So… that is my experience with OAT.* (Woman, late 50s)

This story contains different negative narrations of control – of continued urine testing as the person ages, of restricted take-home arrangements following a positive urine-test for alcohol, and the conflict with adequate palliative care of a husband in pain because of restricted access to painkillers while in OAT. This latter experience is an important reminder that appropriate pain management is required for older people including those in OAT. The following excerpt brings back in the theme of ambivalence, in that whilst testing and control were experienced as unfair and unnecessary, this was tempered later in the interview by a recognition that some people sell their OAT medicine:

Erica: *Those of us who have been in OAT for so many years and live a life of peace and quiet and are just interested in avoiding… in just living a normal life and being allowed our medicines when we wake up in the morning*,* like everyone else who has medicine*,* we have after all patient status and – imagine the fuss if a diabetes patient was told “no no*,* you have to come down to the pharmacy once a week and show us that you take your insulin”* (Woman, 60+).

This story narrates a desire for ‘a normal life’ after ‘so many years’ in OAT contrasted with the experience of supervised intake of prescription opioids. There is a significant challenge here. On an individual level, control and testing can be experienced as restrictive and *ab*normalising, yet the systemic perspective in Norwegian OAT guidelines [11] affirms that the risks of diversion and of overdose still need to be managed (see also [69]). Indeed, Erica herself acknowledged later in the interview that she knew about a few people selling their buprenorphine, earning about 9000NOK a month. There are indications that, within a prison setting, increased emphasis on control may lead to a form of collective resistance and stories of increased diversion [70].

Control was also discussed in the context of age-related somatic problems, including after a diagnosis of severe COPD (chronic obstructive lung disease, a disease which increases in prevalence with age [71]):

Julia: *That phase*,* when I started to get really sick*,* then OAT did a bad job. My GP called a meeting and said*,* “XXX needs peace now. There can’t be anything more now. She has to be allowed to be just a patient now. A normal patient.” But… OAT really didn’t want to back off*,* you know. They nagged*,* but… then my GP banged the table and said*,* ‘XXX must have peace!’ […] So I ended up in there*,* it was a really tough period with oxygen and a wheelchair and. it was really tough to be both an OAT patient and a normal patient. I wish that I was a patient treated holistically.* (Woman, 60+)

This story has many aspects. Julia does some boundary work [72] here, positioning herself and her GP as working together against OAT to achieve treatment as a ‘normal patient’. Even so, Julia evaluates the experience as tough, in part because of experiencing being two types of patient, ‘normal’ and ‘OAT’. This kind of role-splitting is likely to impact more heavily on older OAT patients, in part because of the increased likelihood of comorbidities that come with age, but also because the tension between roles like ‘OAT patient’ and ‘care home inhabitant’ may create additional challenges for older patients should the treatment apparatus not handle such developments appropriately.

### Environmental/contextual factors

#### Housing

Informants had achieved stable independent housing, with some owning outright and others in social housing from the local municipality. Most expressed some satisfaction with their living arrangements, albeit with concerns about the long-term security of renting from the local municipality and the availability of more supported types of accommodation in the future:

Erica: *I am terrified I will like get old and end up in an old people’s home*,* and not get the medical treatment I need. And I think many are the same as time goes on.* (Woman, 60+)

Greta: *I rent from the local municipality*,* a house*,* and that’s partly my own fault*,* because I struggled with my finances when I was using and afterwards. I like it here*,* it’s not that*,* but you want something more secure and your own like.* (Woman, late 50s)

Overall, stable housing seemed to contribute positively to informants’ quality of life, albeit with concern about the future, including a specific concern about access to medication. Such concern is not without foundation: “suitable housing with adequate follow-up was essential in order to provide assistance to nearly every other problem area […] However, there was a lack of such housing for OMT patients in high demand areas in particular” [73]. The excerpt from Greta also mentions financial struggles – we know 83% of opioid patients in Norway are not in education or employment, and 70% have disability or old-age pension as their source of income [74]. The socioeconomic status of OAT patients has the potential to intersect negatively with housing opportunities along with other quality of life domains.

#### Victimisation is not a young person´s problem

We know that, prior to starting OAT, both men and women with opioid use disorders experience victimisation [75, 76]. Even whilst having been in treatment for significant time, female informants told stories of continued victimisation from both physical and sexual violence – as well as the ongoing impact of past traumatic experiences. Regarding the impact of physical violence on current quality of life:

Erica: *I was really badly abused by a partner I had*,* seriously like*,* he tried to kill me. And [pause] yeah*,* it has destroyed my life completely*,* I’m left here now and am totally dependent on assistance to walk and I’ve been in and out of hospital I don’t know how many times*,* it is an incredible number of times [describes specific injuries that create long-term mobility issues]. So I am 100% disabled*,* and I walk with a rollator and crutches and now I have to have a new major operation as well.* (Woman, 60+)

This excerpt is a stark reminder of the impact of intimate partner violence and the ongoing effects such violence has on quality of life – even as people age. The impact is described mainly in terms of consequences for health and mobility, but such events also lead to experiences of stigmatisation:

Frankie: *I am very traumatised because of a lot of very violent relationships*,* but there is no talk of someone like me getting a diagnosis*,* like. No*,* we don’t get that. We aren’t like normal people. We’re apparently something totally different*,* I don’t know. We are treated differently from other people at least.*

[later in the same interview] *I experienced a rape eight months ago and… and then I had a group meeting right afterwards and the*,* the only thing the psychologist*,* she is trained as a psychologist her that I have [as a contact] in OAT*,* the only thing she was concerned about was that I had to begin with urine testing again. Yeah*,* because she thought that: well*,* you have experienced something painful*,* so then you’ll relapse.* (Woman, late 50s)

These stories are important in not only the experiences of sexual and physical violence they recount but also the negative experiences informants relate regarding their treatment afterwards. The first excerpt explicitly engages in boundary-work [72], framing OAT patients as being treated as unlike ‘normal people’ and the second narrates a painful experience of increased control as a response to a traumatic incidence of sexual violence. We see ageing as important here for two reasons. The first is that these stories serve as an important reminder that victimisation can occur at all ages and that treatment personnel should maintain awareness of this possibility when engaging with older patients. Second, and linked to this, is the risk that treatment personnel become desensitized for the stories of violence among patients, perhaps particularly when related by older patients who have been in treatment for a long time. So, treatment personnel need to maintain both awareness of and empathy for such victimisation even as patients age to ensure all who experience traumatic events like sexual violence are met with empathy and support.

#### The time left

A number of informants narrated a sense of having a defined, and often limited, amount of time left:

Erica: *And I know there is one thing that is very important to me*,* which is that I don’t know anyone who is older than – the oldest OAT patient I know is in their early seventies*,* I think. Most die too early. If it isn’t medicines or overdoses and such*,* then it is that the body says stop*,* and therefore it’s like – they say I have gallows humour – now I am in my early 60s and okay*,* then I have ten years left so I have to live as well as I can those ten years*,* and therefore I want it to be as easy as possible.* (Woman, 60+)

Frankie: *I have a sort of goal to live until I am over 80*,* but… I think that then I have 20 years left and if it’s going to be like it is now regarding [OAT]*,* then I will get tired out earlier. I will. But if I can get trust and help*,* without having to battle all the time*,* then I can get 20 years*,* get 20 good years.* (Woman, late 50s)

Harry: *I hope that my partner and I can live 5*,* 6*,* 7 8 more years*,* but I assume that within the next 4–5 years we will have to move into a nursing home or get visits from a home nurse and so on. You notice that when you hit 65 then you start to struggle*,* you know. It isn’t exactly something I’m looking forward to*,* but I’m pretty sure that in a few years we’ll need more help.* (Man, 60+)

These accounts all include informants narrating awareness that they only have a certain amount of time left. There is an element of boundary work here, with the informants framing OAT patients as having shorter life expectancy both explicitly (‘most die too early’) and implicitly (in terms of imposing an expected time limit). The first and second account also position OAT as (potentially) making these final years more difficult – Erica’s reference to ‘as easy as possible’ is in part a point about a desire for reduced control/safety measures from OAT, whilst Frankie explicitly frames OAT as both a negative influence on her quality of life and as potentially shortening her life. These narratives though also show the informants seeking to create meaning by affirming that they want to make the best of the time they have left, albeit that some make a bleaker point:

Bex: *As I get older*,* I understand why people commit suicide.* (Woman, early 50s)

## Concluding discussion

The goal of this article was to explore how older Norwegian OAT patients narrate and make sense of quality of life as they grow older. In starting to address the lack of research on the distinct experiences of older OAT patients and their quality of life, we sought to shed light on the diversity of life experiences and trajectories. Our analysis encompasses both *what* factors our informants narrated as enhancing or diminishing their quality of life and *how* these factors impact on quality of life. This is an important contribution of this kind of fine-grained qualitative analysis. Key factors narrated as enhancing quality of life for our informants were: positive relationships with family and friends, physical activity, overarching and specific help from OAT and stable housing (reinforcing [21, 26]). Factors narrated as diminishing quality of life were loneliness, isolation and bereavement, comorbidities, short-term memory problems, staff turnover, experienced lack of trust and excessive control/safety measures in OAT, and victimisation. Regarding *how* these factors affect life quality, they can impact in a direct manner (e.g. bereavement), in a mutually reinforcing manner (e.g. good family relationships being a source of physical activity, or isolation and a lack of ‘backstage’ leading to volatile interactions with treatment personnel), or they can act to expand or constrain the identity narratives OAT patients experience as more or less available (e.g. wanting to be treated as a ‘normal’ hospital patient but relating an experience of being both a ‘normal’ and ‘OAT’ patient simultaneously). Whilst some informants described OAT as having the capacity to respond with individualised approaches, there is room for improvement here as patients engage with multiple, intersectional challenges that play out across dimensions of age, gender, victimisation, health status and socioeconomic status.

Regarding limitations, the small sample size and single-country geographical focus implies any generalization of findings will necessarily be analytical. Recruiting via user organisations has advantages in helping manage consent and accessing potentially hard-to-reach people, but it also means little control over who volunteers outside the initial inclusion criteria. So, this means the sample could be skewed towards those with particularly positive/negative views of OAT. Nonetheless, the range of views expressed in the interviews suggests those who volunteered reflect the range of OAT-experiences we see in survey data (e.g [35]). We should also acknowledge the experiences of our informants here include time in OAT when it was much more restrictive and controlling – people carry with them such experiences. Regarding gender, the underrepresentation of female SUD patients in research has been long recognized. As set out above, women are in fact over-represented in our sample. Whilst we have used intersectionality to shed light on how multiple factors have a compound effect on quality of life, we lack data on race/ethnicity both specifically in this study and regarding the Norwegian OAT population more broadly. This is a potential avenue for future research, as is taking a specifically gendered approach to understanding how OAT is experienced in Norway.

Returning to our introductory comments on Norway as a critical case, the state’s high capacity and will to help and intervene with its polity has helped keep OAT patients alive (Europe-wide, 28% of OAT patients are aged 50 and over [77], whilst in Norway 44% are aged 50+ [1]). These results do though appear to have come as a result of balancing the needs for safety measures and quality of care that, from one perspective may preserve life, and thus be the prerequisite for the ageing OAT population, but from the individual patient’s perspective may be experienced as a lack of trust, as sometimes acting to marginalise them, and as negatively impacting on their quality of life. Their advancing age and expressed desires for both calm and dignity highlights the need for exploring a new but explicit phase of OAT specifically tailored towards later life stages. Harnessing and directing the state’s capacity towards enhancing dignity for this ageing, often marginalised and increasingly vulnerable group will be increasingly important to enable them to make the most of what they describe as their ‘time left’. In our analysis we showed how issues our informants associated with getting older (e.g. UTIs, drowsiness, memory issues) can be misinterpreted by treatment personnel as symptoms of drug use. In addition, the intersection of isolation and mobility problems creates an experience of lacking agency and of dependence on others – which can in turn lead to experiences of difference from other types of patients and to negative interactions with health personnel.

We see therefore three important, intersecting thematic implications for practice and further research, and one overarching recommendation for an age-informed treatment model. The three thematic implications address memory issues, victimisation and network-building. Memory issues appear particularly important given the narrated impact on people’s lives, including on agentic, quality-of-life-improving behaviours like socialising. Such issues may also impinge on the effectiveness of standardised treatment approaches (e.g. CBT). Treatment personnel also need awareness of potential victimisation amongst older patients and ought to respond in constructive, empathic ways should such violence occur (e.g. via reflective listening – see [78]). Network-building is also a significant issue because of the challenges flowing from bereavement, cutting off drug-using friends/acquaintances and the experience of finding it difficult to build new networks at an older age. These three issues also have the potential to intersect in a negatively reinforcing manner and as such should be prioritised in treatment of older OAT patients. Building on this, we also see potential for the development and introduction of a treatment model/phase specifically for older OAT patients. Such a model should take an age-informed approach to (1) the additional complexity older OAT patients face, (2) the nature of the help and support offered by OAT, and (3) appropriate, ideally lighter-touch safety measures in this final stage of OAT. Such a treatment model would decrease the risk of prolonging of patients’ lives at a too high cost in terms of dignity and life quality. Here there is the potential to further mobilise the experience of older OAT patients to reinforce the possibility of—and deliver on the public sector duty to support—the enhancement of people’s intrinsic capacity across the entire life course.

## Data Availability

The interview data used in this study contain sensitive personal information including health data. As such, the transcripts are not publicly available. All excerpts used in the study have been de-identified. For queries on this please contact John Todd-Kvam, the corresponding author.
